# Rapid, Sensitive, and Selective Quantification of *Bacillus cereus* Spores Using xMAP Technology

**DOI:** 10.3390/microorganisms10071408

**Published:** 2022-07-13

**Authors:** Houman Moteshareie, Walid M. Hassen, Yasmine Dirieh, Emma Groulx, Jan J. Dubowski, Azam F. Tayabali

**Affiliations:** 1 Department of Electrical and Computer Engineering, Interdisciplinary Institute for Technological Innovation (3IT), Université de Sherbrooke, Sherbrooke, QC J1K 2R1, Canada; houman.moteshareie@usherbrooke.ca (H.M.); mohamed.walid.hassen@usherbrooke.ca (W.M.H.); jan.j.dubowski@usherbrooke.ca (J.J.D.); 2 Biotechnology Laboratory, Environmental Health Science and Research Bureau, Healthy Environments and Consumer Safety Branch, Environmental Health Centre, Health Canada, Ottawa, ON K1A 0K9, Canada; yasmine.dirieh@hc-sc.gc.ca (Y.D.); emma.groulx@hc-sc.gc.ca (E.G.)

**Keywords:** recombinant antibody, aptamer, *Bacillus cereus*, spore, xMAP, multiplex

## Abstract

*Bacillus cereus* is a spore-forming ubiquitous bacterium notable as a food poisoning agent. Detection of *B. cereus* spores using selective media is laborious and non-specific. Herein, the quantitative detection of *B. cereus* spores was investigated with commercial antibodies and published aptamer sequences. Several detection reagents were screened for affinity to *Bacillus* collagen-like protein A (BclA), an abundant exosporium glycoprotein. Sensitivity and selectivity toward *B. cereus* spores were tested using immunoassays and multi-analyte profiling (xMAP). A recombinant antibody developed in llama against BclA protein showed *B. cereus* spore selectivity and sensitivity between 10^2^ and 10^5^ spores/mL using xMAP. DNA aptamer sequences demonstrated sensitivity from 10^3^ to 10^7^ spores/mL and no cross-reaction to *B. megaterium* and *B. subtilis*. Selectivity for *B. cereus* spores was also demonstrated in a mixture of several diverse microorganisms and within a food sample with no compromise of sensitivity. As proof of concept for multiplexed measurement of human pathogens, *B. cereus* and three other microorganisms, *E. coli, P. aeruginosa*, and *S. cerevisiae*, were simultaneously detected using xMAP. These data support the development of a rapid, sensitive, and selective system for quantitation of *B. cereus* spores and multiplexed monitoring of human pathogens in complex matrices.

## 1. Introduction

The genus *Bacillus* covers a phenotypically large, heterogeneous collection of Gram-positive, spore-forming, aerobic, or facultative anaerobic bacteria. These organisms exhibit a wide range of physiological capacities for the production of enzymes, antibiotics, and metabolites that allow them to persist in diverse natural environments. As such, *Bacillus* species are frequently used for medical, pharmaceutical, and industrial applications, but they can also be contaminants of plant-based food products [1,2].

The majority of *Bacillus* species rarely cause disease. However, among pathogenic *Bacillus* species, several are genetically related and belong to the *Bacillus cereus* group (BC group). Of these, *Bacillus cereus* causes food poisoning, *Bacillus anthracis* is the etiological agent of anthrax, and *Bacillus thuringiensis* is pathogenic to insect larvae and used as a bioinsecticide. The spores of the BC group are extremely resilient and exhibit resistance to heat, cold, radiation, desiccation, and disinfectants [2].

*B. cereus sensu stricto* (*s.l.*) can replicate and produce toxins in a wide variety of foods and is able to form heat-resistant endospores. Its toxins directly cause food poisoning when viable cells/spores grow in improperly stored foods [3]. However, consumption of as little as 10^3^ viable cells or spores can also cause food poisoning [4]. Ingested spores germinate and produce a number of virulence factors, which can cause diarrhea and vomiting [5,6,7]. Abdominal cramps and watery diarrhea are usually related to four enterotoxins; (1) hemolysin BL (HBL, encoded by hblA, hblC, and hblD), (2) non-hemolytic enterotoxin (NHE, encoded by nheA, nheB, nheC), (3) enterotoxin FM (EntFM, encoded by entFM), and (4) cytotoxin K (CytK, encoded by cytK) [8,9,10,11,12,13]. Vomiting or emesis is caused by a small, heat, and acid-stable cyclic dodecadepsipeptide toxin called cereulide [14,15]. In addition to food poisoning, *B. cereus* is also known to cause serious infections such as pneumonia, endophthalmitis, bacteremia, endocarditis, necrotizing fasciitis, and osteomyelitis [16,17,18].

Despite safety precautions, numerous *B. cereus*-related food poisoning outbreaks have been reported globally in the past decades [19,20,21,22,23]. According to the Centers for Disease Control (CDC), there were 619 confirmed outbreaks of *B. cereus*-related food poisoning from 1998 to 2015, involving 7385 illnesses [24,25]. An estimated 63,400 episodes of *B. cereus*-related illnesses are reported annually in the United States, with many more unreported cases due to the lack of systematic surveillance and low severity of symptoms. Furthermore, Bc-related illnesses are often misdiagnosed as those from *Staphylococcus aureus* or *Clostridium perfringens* infections based on similarity of symptoms [26]. In Europe, it is the second most common cause of foodborne outbreaks after *Staphylococcus aureus* [23]. In 2017, 44% of the reported cases of foodborne outbreaks in France were caused by *B. cereus*. Although mortality is rare, these outbreaks are costly for food and dairy industries and health care systems, as well as economic impacts through employee absenteeism [27]. Nevertheless, *B. cereus* food poisoning can be significantly reduced with frequent, rapid, and cost-effective spore detection methods.

While traditional bacterial detection methods such as colony phenotyping, PCR, or sequencing offer adequate sensitivity [28,29], they are often relatively expensive, labour-intensive, and time-consuming. Nucleic acid-based detection of *B. cereus* spores is complex and requires highly qualified laboratory technicians for both sample preparation and data analysis [29,30,31]. Although xMAP technology is also complex with some similar disadvantages, it can be used for the simultaneous detection of pathogens within a single sample [32]. Furthermore, quantification of *Bacillus* spores from food samples is not easily achieved with nucleic acid-based detection methods due to the hardy nature of endospores, which require significant pre-processing for DNA isolation from food matrices or environmental samples [33]. As such, in many diagnostic protocols, spores are germinated, and cell lysates are extracted from outgrowth cultures [27,34]. This adds to the time and labour necessary for nucleic acid-based detection. In addition, foodborne illnesses are sometimes caused by more than one species, so multiplex assays would offer rapid and sensitive detection [32]. Current PCR systems target a limited number of microorganisms [35,36]. In contrast, the xMAP technology offers well-defined and consistently reproducible assays that can be used to detect multiple foodborne pathogens. This technology uses a magnetic bead-based multiplexed immunoassay system in a microplate format that can simultaneously detect as many as 100 analytes in a single sample (Bio-Rad/Luminex).

In this study, an exosporium protein called *Bacillus* collagen-like A (BclA) was targeted for its antigenicity since it is unique to the BC group. Two classes of antibodies against BclA were applied to develop an xMAP multi-plex assay. Three published DNA aptamers sequences were tested for their capacity to capture BC group spores [37,38,39]. Antibody and aptamer-based assays were demonstrated to detect *B. cereus* spores with high sensitivity in a food sample and in the presence of diverse microorganisms. Furthermore, a multiplex antibody-based assay was developed to selectively and simultaneously detect four different microorganisms (*B. cereus*, *E. coli*, *P. aeruginosa*, and *S. cerevisiae*). This relatively low-cost assay can be adapted to rapidly detect multiple pathogens in a variety of complex matrices.

## 2. Materials and Methods

### 2.1. Bacterial Strains, Plasmids, and Reagents

*Bacilluscereus* (ATCC 14579), *Bacillus megaterium* (ATCC 14581), *Bacillus subtilis* subsp. subtilis (ATCC 6051), *Arthrobacter globiformis* (ATCC 8010), *Pseudomonas fluorescens* (ATCC 13525), *Rhodococcus rhodochrous* (ATCC 53968), and *Pseudomonas aeruginosa* PA01 were obtained from the American Type Culture Collection. Yeast strain BY4743 was a kind gift from Dr. Ashkan Golshani (Golshani laboratory/Carleton University) and used for specificity experiments with xMAP technology. Plasmids were maintained in Escherichia coli DH5α, and recombinant proteins were expressed in *E. coli* BL21 (DE3) (NEB Inc., Ipswich, MA, USA). The BL21 (DE3) was also used in all xMAP experiments with *E. coli*. The microbial mixture contained *B. megaterium*, *B. subtilis*, *A. globiformis*, *P. fluorescens*, *R. rhodochrous*, and *E. coli* BL21 (DE3) strain. A gene encoding *B. cereus* spore BclA protein was cloned into a plasmid (pET151/D-TOPO) (Invitrogen, Waltham, MA, USA). This plasmid was constructed for the expression and isolation of recombinant protein with an N-terminal tag holding the V5 epitope and a hexahistidine tag. Tobacco etch virus (TEV) nuclear-inclusion-a endopeptidase was used to remove the amino-terminal histidine tag. The plasmid also contained an ampicillin-resistant gene that was used as a selectable marker for the isolation of transformed *E. coli* cells.

The following chemical/biological compounds were used: acetone (ACP, Montréal, QC, Canada), 1-Ethyl-3-(3-dimethylaminopropyl)carbodiimide (EDC), OptiClear (National Diagnostics, Mississauga, ON, Canada), ammonium hydroxide 28% (Anachemia, Richmond, BC, Canada), isopropyl alcohol (Fisher Scientific, Ottawa, ON, Canada), anhydrous ethanol (Commercial Alcohols Inc., Brampton, ON, Canada), N-hydroxysuccinimide (NHS), phosphate buffer saline (PBS) (Gibco^TM^, Waltham, MA, USA), ethanolamine (Sigma-Aldrich, Oakville, ON, Canada), Bio-Plex Pro Magnetic COOH Beads (Bio-Rad, Hercules, CA, USA), Bio-Plex Streptavidin-PE (Bio-Rad, Hercules, CA, USA), Bio-Plex^®^ Wash Buffer (Bio-Rad, Hercules, CA, USA), hamster and llama recombinant antibodies (rAb) against BC group BclA protein (Creative Biolabs, Shirley, NY, USA), and thiolated aptamers 1, 2, and 3 (IDT, Kanata, ON, Canada). The food sample was prepared by using 5% GERBER^®^ Stage 1 Rice Baby Cereal. In all experiments, deionized (DI) water with a resistivity of 18.2 MΩ was used.

### 2.2. Microorganism Preparations and Plasmid Miniprep

Bacterial growth was conducted in lysogeny broth (LB) or LB with 2% agar. YP (1% Yeast extract, 2% Peptone) with 2% dextrose as a source of carbohydrates was used as a culture medium for yeast [40]. *B. cereus*, *B. subtitlis,* and *B. megaterium* spores were grown and purified according to Lin et al., 2003 [41] with ~90% purity for *B. cereus* spores and ~70% for the spore of other bacilli, as confirmed by microscopy. *Bacillus* and *E. coli* cells were grown at 37 °C, and recombinant protein expression from a pET151/D-TOPO plasmid in *E. coli* was induced before harvest. All plasmid extractions were performed by using the GeneJET plasmid miniprep kit (Thermofisher, Waltham, MA, USA) following the manufacturer’s instructions.

### 2.3. Purification of Recombinant BclA Protein

Purification of recombinant BclA protein was performed according to Moteshareie et al., 2022 [42], where *N*-terminal histidine-tagged recombinant BclA protein carrying the amino-terminal HIS-tag was purified from plasmid-transformed BL21 (DE3) cells using 3 mL HisPur™ Cobalt Spin Columns (Thermo Fisher Scientific, Waltham, MA, USA). *E. coli* cells were grown in LB at 37 °C to an optical density of 0.4 at 600 nm. After cooling the cultures to 30 °C, recombinant BclA protein expression was induced by 400 μM isopropyl β-d-1-thiogalactopyranoside (IPTG) for 14 h at 30 °C before harvest. Bacteria were sedimented by centrifugation (5000 rpm, 20 min, 4 °C) and re-suspended in binding buffer (40 mM Tris-HCl, pH 7.5, 200 mM NaCl, 10 mM imidazole) supplemented with protease inhibitors and lysozyme (Thermo Fisher Scientific™, Waltham, MA, USA). Bacteria sonicated on ice (2 min with 50% power) in the presence of 25U/mL Pierce Universal Nuclease (Thermo Fisher Scientific™, Waltham, MA, USA). Cell debris was pelleted by centrifugation (15,000 rpm, 20 min, 4 °C). The supernatant was loaded onto a 3 mL column pre-packed with cobalt (Co^2+^) immobilized metal chelate affinity (IMAC) beads (Thermo Fisher Scientific™, Waltham, MA, USA) and pre-equilibrated with binding buffer. Following washing with 10 mL of binding buffer, proteins were eluted with elution buffer (binding buffer plus 250 mM imidazole). Fractions containing the recombinant protein were confirmed by SDS-PAGE (Supplementary Figure S1), pooled, and dialyzed against storage buffer (25 mM Tris-HCl, pH 7.5, 200 mM NaCl). The terminal histidine tag was removed with 3000 U/mg protein of TEV protease (NEB Inc.) incubated at 4 °C for 1 h. Protein concentrations were measured using a NanoDrop at 280 nm (Thermo Fisher Scientific™, Waltham, MA, USA), and the final purified BclA protein was stored in aliquots at −80 °C.

### 2.4. Immunoblotting

Total proteins were purified in the absence of detergent [43]. Briefly, samples containing spores were pelleted by centrifugation and rinsed with 1× PBS. Total protein concentration was measured using the bicinchoninic acid assay (BCA) using protocols from the manufacturer (Thermo Fisher Scientific, Waltham, MA, USA). Equal amounts of protein were electrophoretically separated in 10% polyacrylamide-SDS gels using a Mini-PROTEAN Tetra cell electrophoresis unit (Bio-Rad^®,^ Hercules, CA, USA) [40]. The proteins were transferred to 0.45 μm nitrocellulose (Bio-Rad^®^, Hercules, CA, USA) with a Trans-Blot Semi-Dry Transfer system (Bio-Rad^®^, Hercules, CA, USA). Table 1 shows the tested antibodies. Following transfer, immunoblots were viewed with enhanced chemiluminescence (ECL) substrates (Bio-Rad^®^, Hercules, CA, USA) using a Vilber Lourmat gel doc Fusion FX5-XT (Vilber^®^, Marne-la-Vallee, France). Densitometry was conducted with FUSION FX software (Vilber^®^, Marne-la-Vallee, France), using total protein to normalize measurements. Data represent three separate experiments from three different total protein isolations.

### 2.5. Paratope Blocking Assay

A paratope blocking approach was used to study the accessibility of antigenic target proteins on the surface of *B. cereus* spores [42]. The premise logic of this assay was that pre-incubating a *B. cereus* spore pAb with BclA recombinant protein would block the BclA paratope of the pAbs, resulting in a notable reduction in BclA-associated banding on immunoblots. For this, 10 μg of the isolated recombinant BclA was pre-incubated with 10 μM of pAb1 in 1× PBS (pH 7.5) for 24 h at 4 °C. The pre-incubated antibodies were then used in immunoblotting procedures as previously described [42].

### 2.6. Enzyme-Linked Immunosorbent Assay (ELISA)

A sandwich enzyme-linked immunosorbent assay (ELISA) method was conducted to measure *B. cereus* spores [42]. In summary, maleimide-activated microtiter plates (Thermo Fisher Pierce) were coated with 10 μg/mL capture Abs (mAb2, rAb3, and rAb4) in 200 μL of 1× PBS (pH 7.5) and incubated at 4 °C for 24 h. Wells were washed twice with wash buffer (1× PBS plus 1% Tween) and then blocked at 30 °C for 2 h with blocking buffer containing 200 μL of 5% non-fat dry milk in 1× PBS. Following 3 washes with washing buffer, the wells were incubated with 200 μL of serially diluted spore samples for 2 h at 30 °C. After 3 more washes with washing buffer, the wells were incubated with 10 μg/mL of HRP-conjugated detection Ab (pAb1) in 200 μL of 1× PBS (pH 7.5) for 2 h at 30 °C to detect the presence of spores attached to the capture Abs. Following 3 more washes, each well was incubated with detection reagent 3, 3′, 5, 5′-Tetramethylbenzidine (TMB) (Thermo Fisher Scientific) for 15 min. The reaction was arrested with 100 μL of stopping solution (2 M H_2_SO_4_) to record the optical density at 450 nm. To adapt this assay for aptamer capture reagents, the aptamers had to be modified by 5′ thiolation to facilitate their immobilization in the wells. Wells were coated and incubated with 200 μL/well of each aptamer at 10 μM concentration in 1× PBS (pH 7.5) at 4 °C for 24 h.

### 2.7. xMAP Multiplexing Assay

A multi-analyte profiling (xMAP) technology (Bio-Rad^®^, Hercules, CA, USA) was performed according to the Bio-Rad manuals, guides, and protocols. Briefly, magnetic beads were first coupled with specific capture bioreagents (antibody or aptamer), and a sandwich strategy was performed to detect the microorganisms. For this, analytes were captured with a bead-antibody complex, and a biotinylated antibody (Table 1) conjugated with Bio-Plex Streptavidin-PE was used to detect the presence of the analytes.

#### Magnetic Bead Assembly for Detection by xMAP Technology

Four different Bio-Plex Pro Magnetic COOH Beads (Bio-Rad^®^, Hercules, CA, USA) were coupled with specific capture reagents for each organism using a Bio-Plex Amine Coupling Kit (Bio-Rad^®^, Hercules, CA, USA) according to the manufacturer’s instructions. The coated beads were then treated with either *B. cereus* at different concentrations (10, 10^2^, 10^3^, 10^4^, 10^5^, and 10^6^ cells/mL) alone, in the presence of the five diverse microorganisms (Table 1), or with other pathogens. Biotinylated polyclonal antibodies (Table 1) were used as a secondary antibody for detection. Streptavidin-phycoerythrin (Bio-Rad^®^, Hercules, CA, USA) was conjugated to the biotin of the secondary antibody to generate a quantifiable signal.

### 2.8. Statistical Data Analysis

Statistics to analyze data were performed using Graphpad Prism^TM^ (Graphpad Software, San Diego, CA, USA). Differences in the paratope blocking assay were made using a Student’s *t*-test. Differences in treatments were confirmed using one-way analysis of variance (ANOVA) followed by post-hoc analysis using either Tukey’s or Dunnett’s multiple comparison test. A *p* < 0.05 was considered statistically different.

## 3. Results

### 3.1. Screening of Capture Reagents

#### 3.1.1. Antibody Affinity to *B. cereus* Spore BclA Protein

**Figure 1:**
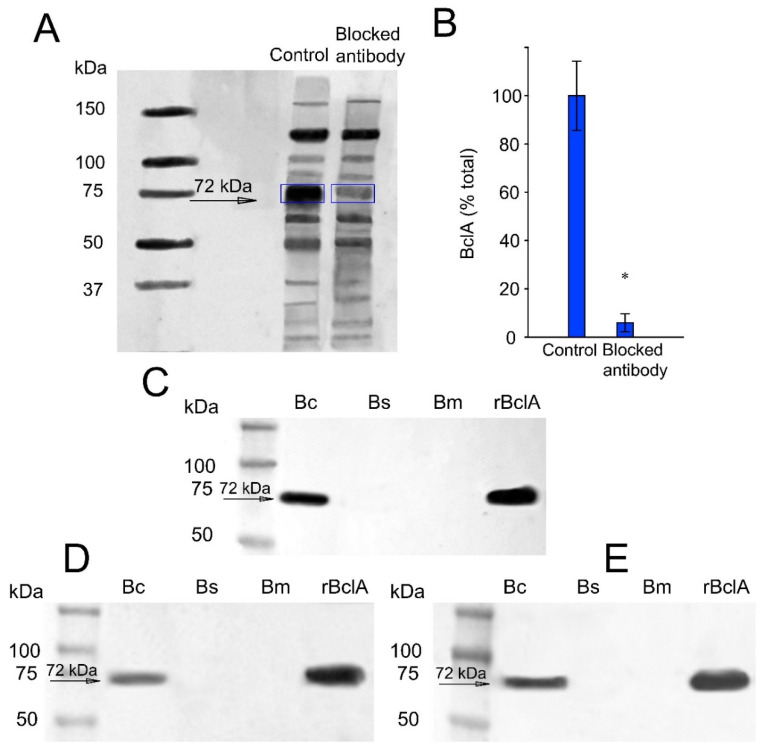
Western blot analysis of antibody interactions with spore coat proteins. (**A**) Competitive paratope inhibition of BclA, using pAb1 (anti-*B. cereus* + *B. subtilis* spores). (**B**) The immunoblot in panel A was quantified by densitometry. Values were normalized to the total concentration of protein per lane. (**C**) Selectivity of the mAb2 (anti-*B.* *anthracis* spores) and binding affinity for isolated rBclA from *E. coli* and two other *Bacillus* species. (**D**) Selectivity of the hamster rAb (rAb3-anti-BclA) toward spore lysate from various *Bacillus* species. (**E**) Selectivity of the llama rAb (rAb4-anti-BclA) with spore lysates from different *Bacillus* species. Wells contained ~25 μg protein lysate and ~20 ng purified rBclA. Panel B: error bars represent standard deviations from three separate experiments. The asterisk indicates a statistically significant difference (*t*-test) between unblocked and blocked pAb (* *p* < 0.005). Abbreviations: Bc, *Bacillus cereus*; Bm, *Bacillus megaterium*; Bs, *Bacillus subtilis*.

To test if the pAb1 could bind spore BclA, a paratope blocking assay was performed (Figure 1A). For this, pAb1 was pre-incubated with rBclA to block its associated paratope, and then the antibody-rBclA complex was used for Western blotting with the entire spore extract. The results showed a significant reduction in the binding of the antibody-rBclA complex to a major spore protein with the same mobility as rBclA compared to unconjugated pAb1. This suggests that immunogenicity to the spore was largely related to BclA (Figure 1A). This was also statistically verified by densitometry (Figure 1B).

The selectivity of mAb2 was tested with spores from *B. cereus*, *B. subtilis*, and *B. megaterium*. As shown in Figure 1C, mAb2 selectively bound *B. cereus* spores, but not those from *B. subtilis* and *B. megaterium*. The peptide detected using this mAb was equivalent in size to that of rBclA at 72 kDa (Figure 1C). Furthermore, two commercially available rAbs derived from hamsters and llamas were tested against *B. cereus* spores (Figure 1D,E). In this experiment, BclA protein of *B. cereus* spores was detected using both commercial rAbs (rAb3 and rAb4). In contrast, neither rAbs demonstrated cross-reactivity with any component of *B. subtilis* or *B. megaterium* spores (Figure 1D,E).

#### 3.1.2. Detection Range of *B. cereus* Spores with rAbs

**Figure 2:**
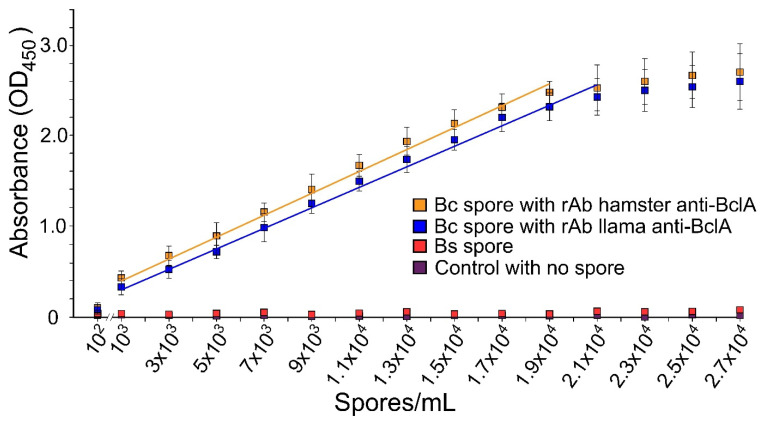
Analysis with ELISA for selectivity and sensitivity of rAbs. A sandwich ELISA strategy was conducted to determine the range of detection sensitivity of the hamster and llama anti-BclA rAbs (rAb3 and rAb4). This experiment was repeated thrice. Error bars represent standard deviations. Trend lines are shown to highlight the linear range of detection. Abbreviations: Bc, *Bacillus cereus*; Bm, *Bacillus megaterium*; Bs, *Bacillus subtilis*.

As both rAbs showed strong selectivity, further experiments to determine their range of detection were conducted by using them as capture reagents in a sandwich ELISA strategy (Figure 2). As indicated in Figure 2, the rAbs showed a detection range between 10^3^ and 1.9 × 10^4^ spores/mL for hamster rAb3 and detection between 10^2^ and 2.1 × 10^4^ spores/mL for llama rAb4. *B. subtilis* spores were used as a control for selectivity in this experiment. As indicated in Figure 2, neither rAbs showed any significant cross-reaction to *B. subtilis* spores nor to vegetative cells (data not presented).

### 3.2. Detection of B. cereus Spores with Aptamers

#### 3.2.1. Selectivity of Detection with ELISA

**Figure 3:**
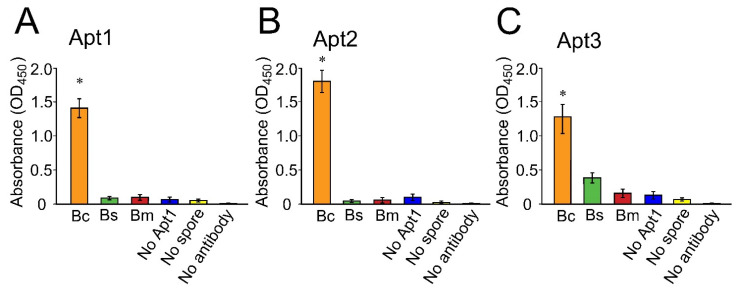
Binding affinity of three aptamers to different *Bacillus* spores at 10^5^ spores/mL using a sandwich ELISA strategy. (**A**) Apt1, selected against *B. thuringiensis* spores. (**B**) Apt2, selected against *B. anthracis* spores. (**C**) Apt3, selected against *B. anthracis* spores. All three aptamers showed binding affinity to *B. cereus* spores. This experiment was repeated thrice. Error bars represent standard deviations. Asterisks indicate statistically significant differences with non-BC group spores or controls and were determined with one-way ANOVA followed by Tukey post-hoc analysis (* *p* < 0.005). Abbreviations: Bc, *Bacillus cereus*; Bm, *Bacillus megaterium*; Bs, *Bacillus subtilis*.

Three assorted DNA aptamers were selected from the existing literature (Table 1) to investigate their potential to be used as capture reagents for the detection of *B. cereus* spores. An aptamer-antibody sandwich ELISA strategy was developed, in which aptamers were immobilized on ELISA plates to capture *B. cereus* spores, and a biotinylated commercial Ab against *B. cereus* spores was used to detect the presence of the captured spores. Figure 3 shows that all aptamers strongly bound to *B. cereus* spores. Apt2, which was developed against *B. anthracis* spores, demonstrated the strongest binding affinity for *B. cereus* spores (Figure 3B).

Apt2 and Apt3 developed against *B. anthracis* spores also demonstrated strong binding affinities for *B. cereus* spores (Figure 3B,C). Apt2 showed no detectable signal for either *B. subtilis* or *B. megaterium* (Figure 3B). However, minimal reaction to the spores of *B. subtilis* or *B. megaterium* was observed, indicating a lower level of selectivity with non-BC group *Bacillus* spores compared to Apt1 and Apt2 (Figure 3C).

#### 3.2.2. Range of Detection with ELISA

**Figure 4:**
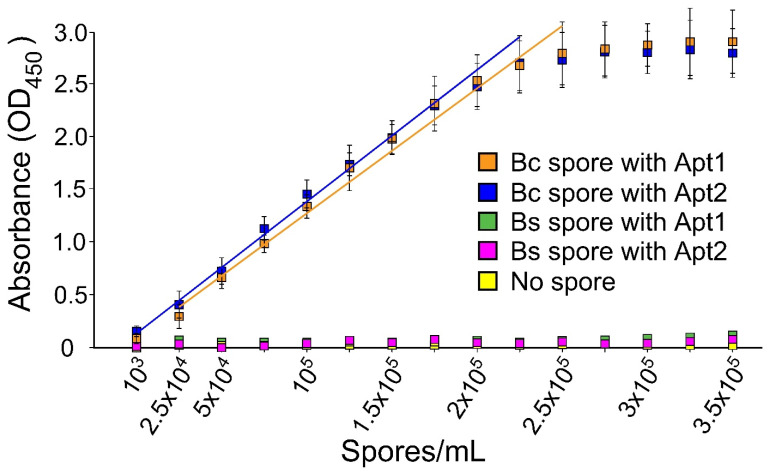
Range of detection of aptamers using ELISA. Detection with Apt1 and Apt2. This experiment was repeated thrice. Error bars represent standard deviations. Trend lines are shown to highlight the linear range of detection. Abbreviations: Bc, *Bacillus cereus*; Bs, *Bacillus subtilis*.

The aptamer-antibody sandwich ELISA method was used to investigate the concentration range of detection of *B. cereus* spores. Following optimization of parameters involved in this strategy, such as capture aptamer and detection antibody concentrations, Apt1 showed a detection range between 10^3^ and 2.25 × 10^5^ spores/mL, and Apt2 showed a detection range from 10^4^ to 2.5 × 10^5^ spores/mL (Figure 4).

**Figure 5:**
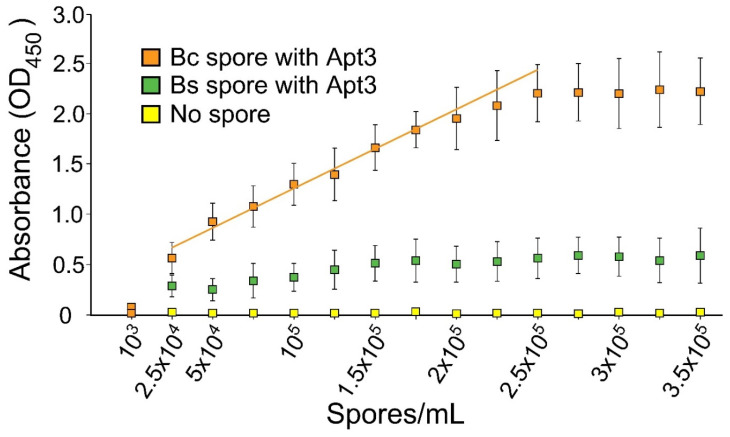
Range of detection of Apt3 using ELISA. This experiment was repeated thrice. Error bars represent standard deviations. A trend line is shown to highlight the linear range of detection. Abbreviations: Bc, *Bacillus cereus*; Bs, *Bacillus subtilis*.

Apt3 also detected *B. cereus* spores at a similar range as Apt1, but Figure 3C shows that it also non-specifically bound reaction to *B. subtilis* spores (Figure 4). The low cross-reaction signal from increasing concentrations of *B. subtilis* is also illustrated in Figure 5.

### 3.3. Detection with xMAP Technology

#### 3.3.1. Antibody-Based Detection of *B. cereus* Spores

**Figure 6:**
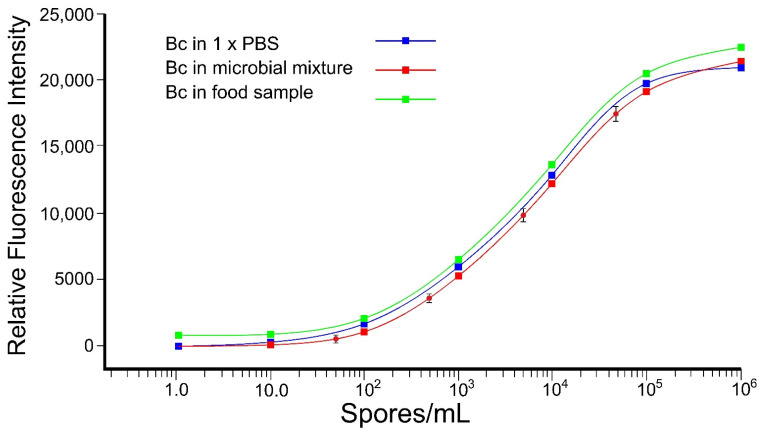
Range of detection using xMAP system. Detection of *B. cereus* (Bc) spore lysate with llama rAb4 in 1× PBS (blue curve), in food sample/5% baby cereal (green curve) and the presence of 6 different microorganisms as a microbial mixture in 1× PBS (red curve). This experiment was performed in triplicate, and no significant difference was observed. The error bars represent standard deviations for tested samples (red dots) at 50, 5 × 10^2^, 5 × 10^3^, and 5 × 10^4^ spores/mL.

The xMAP technology was paired with llama rAb to detect *B. cereus* spores. Initial experiments were performed with *B. cereus* spore lysate in 1× PBS buffer (Figure 6). Followup experiments were performed in the presence of six other microorganisms to test for cross-reactivity and sensitivity in the presence of a microbial mixture (Figure 6). Different concentrations of spores at 50, 5 × 10^2^, 5 × 10^3^, and 5 × 10^4^ spores/mL were used to test the consistency of the standard curve under these conditions. Results showed a range of detection between 10^2^ spores/mL and 10^5^ spores/mL with a low standard deviation for the four tested spore concentrations (Figure 6). As indicated in Figure 6, the standard curves were similar for the detection of *B. cereus* spores alone compared to *B. cereus* in the presence of other microorganisms.

#### 3.3.2. Aptamer-Based Detection of *B. cereus* Spores

**Figure 7:**
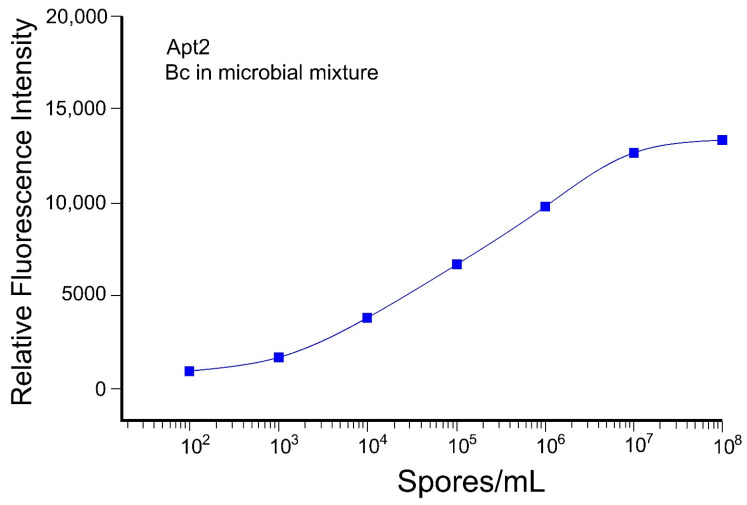
Range of detection using xMAP system. Detection of *B. cereus* (Bc) spores with Apt2 in the presence of 6 different biotechnology microorganisms as a microbial mixture. This experiment was performed in triplicate, and no significant difference was observed.

The possibility of using an aptamer with xMAP technology was tested. Due to its higher sensitivity (Figure 3B), Apt2 was used for this experiment. This aptamer was developed against *B. anthracis* spores and successfully detected *B. cereus* spores with ELISA. Using xMAP technology and pairing Apt2 with the magnetic beads, *B. cereus* spores were successfully detected in a microbial mixture of six other microorganisms (Figure 7), with a range of detection between 10^3^ and 10^7^ spore/mL (Figure 7). This experiment was carried out with total spore lysate, and lower performance may be due to the fact that aptamers in this study were developed using the entire spore.

#### 3.3.3. Multi-Analyte Detection of Four Different Microorganisms

**Figure 8:**
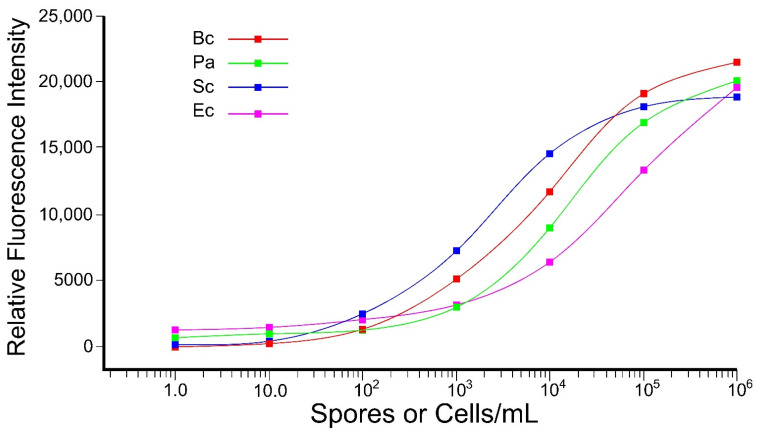
Multi-analyte detection using xMAP system. Antibody-based detection of *B. cereus* (Bc; rAb4) spore lysate along with *P. aeruginosa* (Pa; mAb5), *S. cerevisiae* (Sc; mAb6), and *E. coli* (Ec; mAb7) cell lysates in the presence of other microorganisms as a microbial mixture. This experiment was performed in triplicate, and no significant difference was observed.

Commercial antibodies were paired with different magnetic beads for simultaneous multi-analyte detection of four different microorganisms in the same sample. The antibodies were claimed to be selective to *P. aeruginosa*, *S. cerevisiae,* and *E. coli* (Table 1), but a comprehensive evaluation of specificity was not conducted in this study. All four organisms demonstrated acceptable detection ranges, with minimal cross-reactivity to the microorganisms (Figure 8). The rAb retained its selectivity and sensitivity between 10^2^ and 10^5^ spores/mL during multi-analyte detection. *P. aeruginosa* and *E. coli* showed a range of detection between 10^3^ and 10^5^ cfu/mL. *S. cerevisiae* showed sensitive detection between 10^2^ and 10^4^ cfu/mL. Despite some cross-reactivity, *E. coli* could be detected between 10^3^ and 10^6^ cfu/mL (Figure 8).

## 4. Discussion

A rapid, sensitive, and selective detection system was developed for *B. cereus* spores, which are significant causes of health and economic impacts. Control measures should be focused on spore detection since they are the environmentally stable form of the bacterium that germinates and produce toxins in food matrices. The xMAP system was demonstrated to be robust and amenable to simultaneous detection of several pathogens without interference from a model food matrix of other diverse microorganisms. Although more experiments with other food poisoning pathogens could further validate the use of xMAP technology in the food sector, a proof of concept for multiplex measurement was demonstrated by detecting three different bacteria and yeast.

Due to the strong antigenicity of BclA protein [42], initial efforts for *B. cereus* spore detection focused on the BclA protein. This protein is a hairpin-shaped trimeric glycoprotein that is found in abundance on the exosporium layer of BC group spores [44]. Recombinant BclA protein was synthesized and purified to test affinity to a pAb that was developed against *B. cereus* spores in rabbits (Figure 1A). The pAb was used to determine the antigenicity and accessibility of BclA protein as a target for spore detection. Several mAbs were demonstrated to provide increased selectivity compared to pAbs (data not shown). As indicated in Figure 1C, the strong relatedness of BC group members was exploited to demonstrate that a mAb (mAb2) developed against B. anthracis spores (Table 1) could also be selective for B. cereus spores. Not unexpectedly, the spore protein recognized by the mAb was identical in migratory molecular weight to that of both spore-associated and purified BclA (Figure 1C). The mAb could also successfully detect the recombinant purified BclA, which confirmed its strong antigenicity. Although the results showed selectivity for the mAb with strong recognition of BclA protein, the large-scale production cost, poor clonal reproducibility, and problems with long-term maintenance of mAbs make them inferior compared to rAbs [45,46]. Therefore, two specific rAbs developed in hamster and llama (Table 1) against purified BclA protein were tested (Figure 1D,E). These results indicated that there was no significant cross-reaction with *B. subtilis* or *B. megaterium*, supporting BC group specificity of BclA protein and selectivity of rAbs developed against BclA. Both rAbs were tested for their range of detection with a sandwich ELISA technique to determine their sensitivity for subsequent application using the xMAP strategy. The sensitivity of rAbs with ELISA was shown to be as low as 10^3^ to 2.1 × 10^4^ spores/mL (Figure 2). It should be noted that although the selectivity of BclA rAbs against *B. subtilis* and *B. megaterium* spores demonstrated that they were highly specific for *B. cereus* spores, investigation with other microorganisms (pathogenic or non-pathogenic) that may be present in food and dairy products should be performed to determine any potential cross-reaction.

The rAbs surely provide an excellent source of capture bioreagent, but initial production may be costly. As an alternative, DNA-based aptamers were tested for their high specificity and affinity to capture BC group spores (Table 1). The application of aptamers in magnetic bead-based assays and xMAP technology has been previously illustrated in multiple studies [47,48,49]. In this study, three published aptamer sequences (Table 1) were tested for their ability to capture *B. cereus* spores. As indicated in Figure 3A,B, two of the three aptamers produced no significant signal for *B. subtilis* and *B. megaterium*; however, Apt3 showed small cross-reactivity with *B. subtilis* and *B. megaterium* (Figure 3C and Figure 5). All three aptamers demonstrated a relatively wide range of detection between 10^3^ and 2.25 × 10^5^ spores/mL (Figure 4). The application of Apt1 for the detection of *B. thuringiensis* at 10^3^ spores/mL has recently been reported with a digital photocorrosion biosensor [42].

The xMAP technology is applicable for multiplex detection of different analytes within a single complex sample. xMAP multiplex assays are currently available in various nucleic acid and immunoassay formats, enabling simultaneous detection and typing of pathogenic viruses, bacteria, parasites, and fungi and also antigen or antibody interception [32,50,51]. As an open architecture platform in the xMAP technology, magnetic microsphere sets that are internally labelled with two spectrally different fluorophores can be paired with selective and sensitive capture molecules such as antibodies [32] and aptamers [49,50] for the detection of multiple foodborne pathogens, which is significantly beneficial for food and dairy industries. This technology could dramatically increase the flexibility of pathogen detection in food products [32,50,51,52].

In this study, BclA llama rAb was paired with Bio-Plex Pro Magnetic COOH Beads and then tested for their ability to capture *B. cereus* spores in the absence and presence of other microorganisms (i.e., pathogens or microorganisms used in biotechnology) (Figure 6). When detection of *B. cereus* was performed in baby cereal as a model complex food matrix, no interference was observed (Figure 6). After optimization of all parameters involved in a sandwich immunoassay with xMAP technology using rAb, the range of detection was improved compared to ELISA to ~10^2^ spores/mL. The results demonstrated both sensitivity and selectivity with xMAP when paired with rAb to detect *B. cereus* spores in both pristine conditions with PBS buffer and in the presence of other microorganisms (Figure 6). These results showed negligible interference from PBS alone, food sample, or other microorganisms (Figure 6). In addition, this suggests high selectivity in the microbial mixture for llama rAb developed using BclA protein.

The possibility of using an aptamer conjugated to magnetic beads was tested for the detection of *B. cereus* spores in a microbial mixture. Using this strategy, *B. cereus* spores were successfully detected in the presence of other microorganisms, and sensitivity was improved by ~10-fold following optimization (Figure 7). The inconsequential presence of other microorganisms and low background signal demonstrated that Apt2 was highly selective (Figure 7). Unfortunately, due to the limited availability of known selective aptamer sequences, not all microorganisms may be analyzed by multiplex analysis. The availability of aptamer with strong affinity and selectivity is an obvious limitation for their widespread use as capture reagents for all pathogens, but the possibility of multiplex sensing using a combination of Abs and aptamers can be explored.

Although both Apt2 and llama rAb showed a wide range of detection for *B. cereus* spore, the rAb demonstrated more sensitivity and a lower range of detection at 10^2^ spores/mL versus 10^3^ spores/mL for Apt2. However, Apt2 was able to detect ~100-times more spores compared to rAbs (Figure 6 and Figure 7). It is possible that the smaller size of aptamers permitted greater immobilization on the spore surface, leading to less stearic interference, higher signal, and more sensitivity. Since 10^3^ spores/g or spores/mL of food or dairy products is sufficient for infection in humans, the lower detection limit of 10^2^ spores/mL is in the appropriate range to serve as a screen for contaminated foods. In case higher sensitivity is required, samples can be filtered and further condensed [53,54]. Nevertheless, improved sensitivity compared to ELISA was observed, which may be due to a combination of instrumentation interpolations for the emission signal, and better accessibility of the suspended capture reagent-coupled microspheres compared to immobilized capture reagents on the ELISA plate surface.

Using xMAP technology with bimolecular quantitative detection of *B. cereus* spores is a novel concept demonstrating attractive sensitivity for analysis of starchy foods and dairy samples. Other sensitive methods have been developed, such as quantitative real-time PCR and biosensing devices. These systems may also quantify *B. cereus* at 10^2^ bacteria or spores/mL, comparable to the xMAP strategy; however, the latter has the superior advantage of multiplexing [55,56,57,58]. The xMAP technology has been successfully developed for multiplex detection and quantification of foodborne pathogens with an adequate range of sensitivity based on the infectious dose of the pathogen following human consumption [50,51,52].

As a proof of concept, three diverse organisms (*P. aeruginosa*, *S. cerevisiae*, and *E. coli*) were selected based on the availability of commercial monoclonal-specific antibodies. Following antibody-magnetic bead coupling, the sensitivity of each was tested using xMAP technology (Figure 8). Standard curves for each of the three other microorganisms were achieved with detection ranges from 10^3^ to 10^5^ cells/mL for *P. aeruginosa* and *S. cerevisiae*, whereas *E. coli* showed sensitivity up to 10^6^ cells/mL (Figure 8).

## 5. Concluding Remarks

The ability of several commercially available antibodies and published DNA aptamer sequences to detect *B. cereus* spores was investigated. Based on the results of immunoassays (ELISA and Western blotting), rAbs generated in hamsters and llamas and two DNA aptamers have been demonstrated to be specific and sensitive for *B. cereus* spore detection. Compared to ELISA, the xMAP approach improved the detection range and could be used for the detection of *B. cereus* spores alone in a food sample and in the presence of a mixture of diverse organisms. Most importantly, xMAP technology showed utility as a multiplexing tool that can be developed for selective detection of pathogens in the food and dairy industry. This technique was also successfully demonstrated with aptamers. Collectively, these results indicate that the benefits of using aptamer-based detection can also be extended to other capture reagent-based detection methods such as biosensing devices.

## Figures and Tables

**Table 1:** Capture reagents used in this study.

Number	Capture Reagents	Description
pAb1	Polyclonal Antibody: PA1-73114 (Thermo Fisher Scientific, Waltham, MA, USA)	Rabbit anti-*B. cereus* + *B. subtilis* spores
mAb2	Monoclonal Antibody: G46D (Thermo Fisher Scientific, Waltham, MA, USA)	Mouse anti-*B. anthracis* spores
rAb3	Recombinant Antibody: A4D1 (Creative Biolabs, Inc., Shirley, NY, USA)	Hamster anti-BclA protein (hamster ovary cells (CHO))
rAb4	Recombinant Antibody: A5 (Creative Biolabs, Inc., Shirley, NY, USA)	Llama anti-BclA protein
mAb5	Monoclonal anti-*P. aeruginosa* antibody: ab35835 (Abcam, Inc., Cambridge, UK)	Mouse anti-*P. aeruginosa* outer membrane protein (clone B11)
mAb6	Monoclonal anti-*S. cerevisiae* PGK1 antibody: ab113687 (Abcam, Inc., Cambridge, UK)	Mouse anti-*S. cerevisiae* PGK1 protein (clone 22C5D8)
mAb7	Monoclonal anti-*E. coli* antibody: ab137967 (Abcam, Inc., Cambridge, UK)	Rabbit anti-*E. coli* outer membrane protein
Apt1	DNA aptamer #1 [37]	Developed against Bt spores (61 bases). Modified by 5′ thiolation.
Apt2	DNA aptamer #2 [39]	Developed against *B. anthracis* spores (72 bases). Modified by 5′ thiolation.
Apt3	DNA aptamer #3 [38]	Developed against *B. anthracis* spores (80 bases). Modified by 5′ thiolation.

## Supplementary Materials

The following supporting information can be downloaded at: https://www.mdpi.com/article/10.3390/microorganisms10071408/s1, Figure S1: Recombinant BclA protein, purified from *E. coli* and tested by SDS-PAGE and Coomassie Blue staining.
