# Supplementary material for: Rapid, Sensitive, and Selective Quantification of Bacillus cereus Spores Using xMAP Technology
Source: Microorganisms. 2022 Jul 13;10(7):1408. doi: 10.3390/microorganisms10071408 (PMC9319878; doi:10.3390/microorganisms10071408)
Supplement: Supplementary file 1 [file microorganisms-10-01408-s001.zip › microorganisms-1787991-supplementary.pdf]

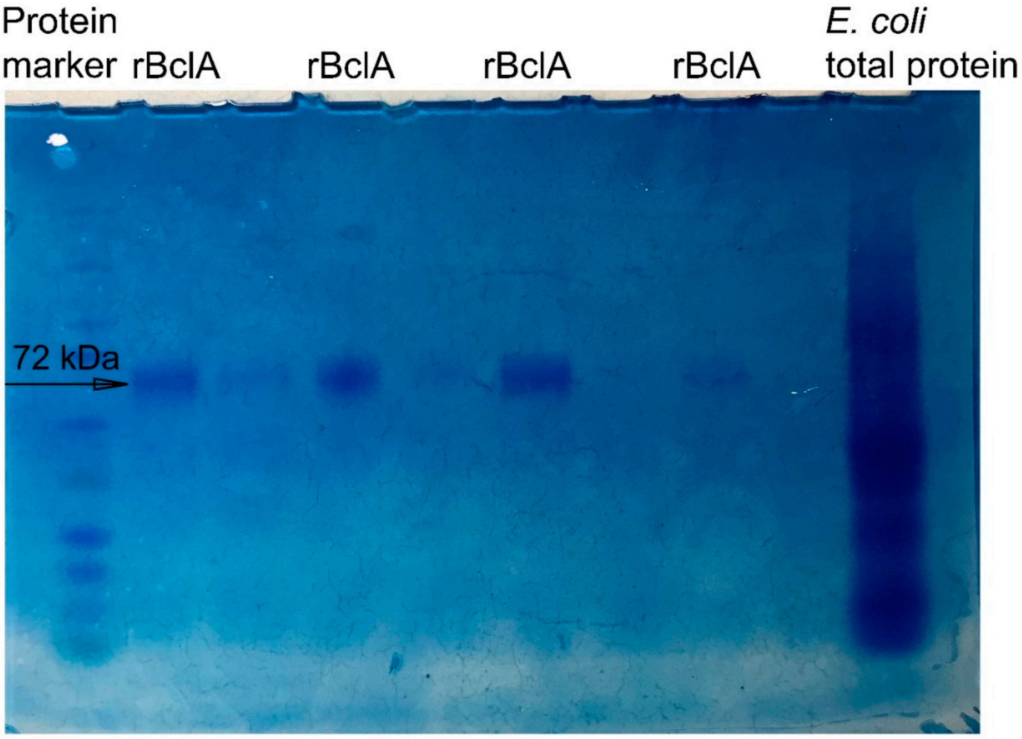

Figure S1. Recombinant BclA protein, purified from *E. coli* and tested by SDS-PAGE and Coomassie Blue staining.
